# ADP-Ribosylation Factor 1 Regulates Proliferation, Migration, and Fusion in Early Stage of Osteoclast Differentiation

**DOI:** 10.3390/ijms161226168

**Published:** 2015-12-09

**Authors:** Min Jae Kim, Hyunsoo Kim, Seoung Hoon Lee, Dong Ryun Gu, Soo Young Lee, Kyunghee Lee, Daewon Jeong

**Affiliations:** 1 Department of Microbiology, Laboratory of Bone Metabolism and Control, Yeungnam University College of Medicine, Daegu 705-717, Korea; t2phage@ynu.ac.kr (M.J.K.); hskim75@ynu.ac.kr (H.K.); 2 Department of Oral Microbiology and Immunology, College of Dentistry, Wonkwang University, Iksan 570-749, Korea; leesh2@wku.ac.kr; 3 Center for Metabolic Function Regulation (CMFR), Wonkwang University School of Medicine, Iksan 570-749, Korea; mrwonsin@naver.com; 4 Department of Life Science and Research Center for Cellular Homeostasis, Ewha Womans University, Seoul 120-750, Korea; leesy@ewha.ac.kr

**Keywords:** ADP-ribosylation factor 1, osteoclast differentiation, osteoclast fusion

## Abstract

Small G-protein adenosine diphosphate (ADP)-ribosylation factors (ARFs) regulate a variety of cellular functions, including actin cytoskeleton remodeling, plasma membrane reorganization, and vesicular transport. Here, we propose the functional roles of ARF1 in multiple stages of osteoclast differentiation. ARF1 was upregulated during receptor activator of nuclear factor kappa-B ligand (RANKL)-induced osteoclast differentiation and transiently activated in an initial stage of their differentiation. Differentiation of ARF1-deficient osteoclast precursors into mature osteoclasts temporarily increased in pre-maturation stage of osteoclasts followed by reduced formation of mature osteoclasts, indicating that ARF1 regulates the osteoclastogenic process. ARF1 deficiency resulted in reduced osteoclast precursor proliferation and migration as well as increasing cell-cell fusion. In addition, ARF1 silencing downregulated c-Jun *N*-terminal kinase (JNK), Akt, osteopontin, and macrophage colony-stimulating factor (M-CSF)-receptor c-Fms as well as upregulating several fusion-related genes including CD44, CD47, E-cadherin, and meltrin-α. Collectively, we showed that ARF1 stimulated proliferation and migration of osteoclast precursors while suppressing their fusion, suggesting that ARF1 may be a plausible inter-player that mediates the transition to osteoclast fusion at multiple steps during osteoclast differentiation

## 1. Introduction

Bone remodeling is an active and dynamic process that involves the coordinated actions of bone formation by osteoblasts and bone resorption by osteoclasts. Osteoclastogenesis reportedly involves a series of regulatory steps such as proliferation and survival of progenitors, differentiation into mononuclear pre-osteoclasts, fusion into mature multinucleated osteoclasts, and activation of osteoclastic bone resorption [1]. The earliest event of osteoclastogenesis is hematopoietic stem cell commitment to the monocyte/macrophage lineage by the transcription factor PU.1, which induces expression of c-Fms, a receptor for M-CSF [2,3]. In the osteoclast precursor stage, activation of M-CSF/c-Fms signaling promotes proliferation of bone marrow-derived macrophages (BMMs), which are osteoclast precursors [1,4]. Consecutively, binding of RANKL to its receptor RANK recruits tumor necrosis factor (TNF) receptor-associated factor 6 (TRAF6), a key adaptor protein [5], and triggers downstream signal molecules for the activation of NF-κB, AP-1, and mitogen-activated protein kinases (MAPKs) [6,7,8]. RANKL also activates nuclear factor of activated T-cells c1 (NFATc1), a master regulator of osteoclast development, through calcium signaling [9,10]. Among multiple steps during osteoclast differentiation, osteoclast precursor fusion is a complicated process including cell adherence to the bone surface, migration, and cell-cell contact as well as being an essential step in forming multinucleated mature osteoclasts via induction of a fusion-competent state [11].

A proper balance between proliferation and differentiation of osteoclast precursors is critical for osteoclastogenesis. First, monocyte/macrophage precursors undergo rapid cell division and then differentiate into tartrate-resistant acid phosphatase (TRAP)-positive mononuclear pre-osteoclasts. In the next stage, fusion of mononuclear pre-osteoclasts into mature multinucleated osteoclasts occurs and osteoclastic bone resorption is activated. Cellular proliferation and differentiation need to be finely tuned and tightly regulated in time. Fusion or maturation of osteoclasts should not occur during the first stage of proliferation. During osteoclast fusion or osteoclastic bone resorption, inhibition of cell proliferation and arrest of cell cycle progression are required. RANKL has been reported to suppress cell proliferation [12] as well as stimulating cell cycle withdrawal through induction of the cyclin-dependent kinase inhibitors p27KIP1 and p21CIP1 [13]. Thus, arrest of osteoclast precursor proliferation after osteoclast fusion is believed to be important to mediate the following propagation of osteoclast differentiation.

ADP-ribosylation factors (ARFs) are members of the Ras superfamily of small guanosine triphosphatases (GTPases) and regulate various cellular processes, including membrane trafficking and actin cytoskeleton remodeling [14,15]. There are six ARF proteins in mammals, which can be divided into three groups according to their amino acid sequences: class I (ARF1, ARF2, and ARF3), class II (ARF4 and ARF5), and class III (ARF6) [16]. Although ARF6 is required for podosome-rich sealing zone formation [17] and myoblast fusion [18], and ARF1 has been reported to be involved in vesicle formation and regulation of cell proliferation and migration [19,20], little is known about the role of ARF1 in bone-resorbing osteoclasts. In the present study, we investigated ARF1 function in the context of regulation of proliferation, migration, and fusion in osteoclast precursors.

## 2. Results

### 2.1. ARF1 Regulates Maturation Step of Osteoclast Differentiation

**Figure 1:**
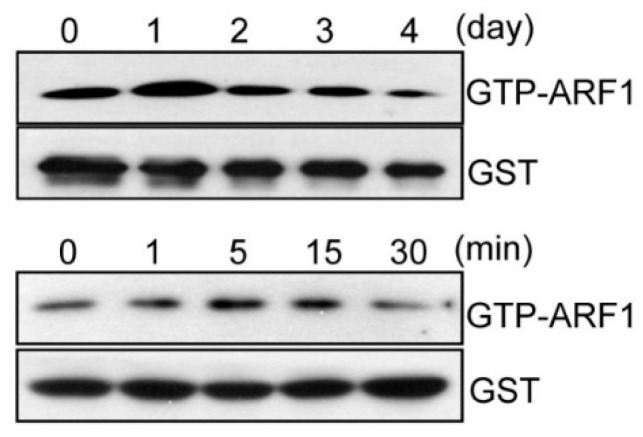
Changes in activity of ADP-ribosylation factor 1 (ARF1) during osteoclast differentiation. ARF1 activity was measured by pull-down assays and immunoblot analysis. Level of guanosine triphosphate (GTP)-bound ARF1 was determined after osteoclast differentiation (**upper** panel) and after transient stimulation by RANKL (**lower** panel). Glutathione S-transferase (GST) was used as a loading control.

**Figure 2:**
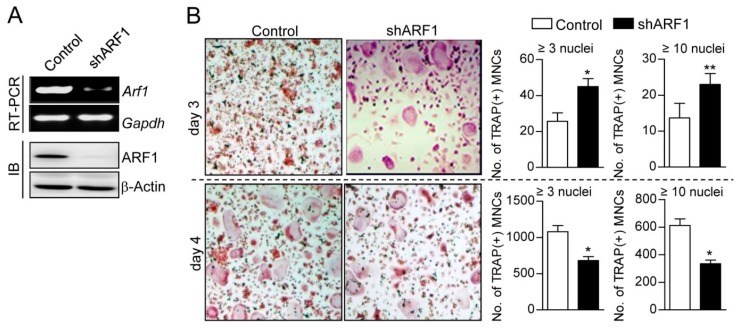
Effect of ARF1 depletion on osteoclast differentiation. (**A**) Osteoclast precursors were infected with shRNA lentiviral particles targeting mouse ARF1 or pLKO.1-puro empty control virus particles. After puromycin selection for 2 days, ARF1 knockdown was confirmed using RT-PCR and immunoblot assay (IB). Glyceraldehyde 3-phosphate dehydrogenase (Gapdh) and β-actin were used as loading controls; (**B**) ARF1-depleted cells with 70% confluence (2 × 10^4^ cells/well) were cultured in 48-well plates and incubated in the presence of M-CSF (30 ng/mL) and RANKL (100 ng/mL) for 3 or 4 days. Tartrate resistant acid phosphatase (TRAP)-positive multinucleated cells [TRAP(+) MNCs] representing more than 3 or 10 nuclei were counted and photographed using a light microscope. The experimental data are expressed as mean ± SD (*n* = 3) and the results shown are representative for at least three independent experiments. * *p* < 0.01, ** *p* < 0.05.

To investigate whether or not ARF1 participates in osteoclast differentiation, we first examined changes in ARF1 activity during osteoclastogenesis. ARF1 showed peak activity on day 1 of RANKL-induced osteoclast differentiation and transiently activated after short-term exposure to RANKL (Figure 1). The mRNA and protein expression levels of ARF1 gradually increased during osteoclast differentiation (Figure S1). To further explore the role of ARF1 in osteoclast differentiation, knockdown of ARF1 was performed using lentiviral-delivered short hairpin RNAs (shRNAs) (Figure S2A). Stable knockdown of ARF1 was maintained at both mRNA and protein levels in osteoclast precursors and mature osteoclasts (Figure 2A and Figure S2B). As shown in Figure 2B and Figure S2C, ARF1 knockdown increased formation of TRAP-positive multinucleated cells on day 3 of osteoclast differentiation but reduced the number of mature osteoclasts on day 4 post-osteoclastogenesis, indicating that ARF1 plays different functional roles in various stages of osteoclast differentiation. Together, such a result suggests that RANKL-induced ARF1 upregulation may be involved in the stages before and after osteoclast maturation. 

### 2.2. ARF1 Deficiency Suppresses Osteoclast Precursor Proliferation and Migration as Well as Potentiates Osteoclast Fusion

**Figure 3:**
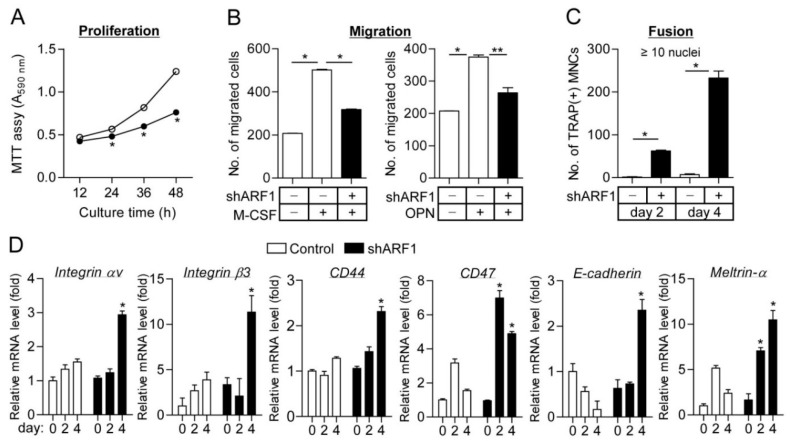
Proliferation, migration, and fusion assay in ARF1-deficient cells. (**A**) Osteoclast precursors infected with ARF1 shRNA lentiviral particles (closed circles) or pLKO.1-puro empty control particles (open circles) were cultured with M-CSF (30 ng/mL) for the indicated times, followed by colorimetric MTT assay; (**B**) Cell migration was determined using the Transwell migration chambers as described under “Materials and methods”. Osteoclast precursors were seeded onto the upper chamber, and M-CSF (30 ng/mL) or osteopontin (OPN, 100 ng/mL) was added to the lower chamber. The number of migrated cells was counted under a light microscope; (**C**) Cell fusion assay. BMMs with 100% confluence were treated with M-CSF (30 ng/mL) and RANKL (100 ng/mL) for 2 or 4 days and then stained for TRAP. TRAP(+) MNCs with more than 10 nuclei were counted using a light microscope. As in (**A**–**C**), data are mean ± SD (*n* = 3). * *p* < 0.01, ** *p* < 0.05; (**D**) Expression levels of fusion-related genes, including αvβ3 integrin, CD44, CD47, E-cadherin, and Meltrin-α, during osteoclast differentiation were analyzed using quantitative real-time PCR. The quantitative data between groups were analyzed by one-way ANOVA comparison. * *p* < 0.01.

In order to verify that ARF1 is connected with osteoclastogenesis, we further observed a definite role for ARF1 in multiple stages of osteoclast differentiation, including proliferation, migration, and fusion. ARF1 silencing resulted in significant reduction of proliferation and migration in osteoclast precursors (Figure 3A,B and Figure S2D), indicating that ARF1 plays an intrinsic role in promoting cell proliferation and migration during osteoclast differentiation. Cell fusion assay of osteoclast precursors showed that shRNA-mediated depletion of ARF1 induced marked elevation of TRAP-positive multinucleated cell formation (Figure 3C). This result is supported by data showing that expression of osteoclast fusion-related genes, including CD44, CD47, E-cadherin, meltrin-α, and αvβ3 integrin, was remarkably upregulated in the maturation stage of osteoclast precursors infected with ARF1 shRNA virus compared to the control group (Figure 3D). Other genes related to osteoclast fusion such as *Atp6v0d2*, *DC-STAMP*, *OC-STAMP*, *MFR*, *CD9*, and *FAK* were not significantly altered by ARF1 depletion (Figure S3). These findings indicate that ARF1 allows osteoclast precursor proliferation and migration in the preceding step of osteoclast fusion and negatively regulates the fusion of mononuclear osteoclast precursor to maintain proliferation and migration in the early stages of osteoclast differentiation.

### 2.3. ARF1 Regulates Diverse Osteoclastogenic Signaling Pathways

**Figure 4:**
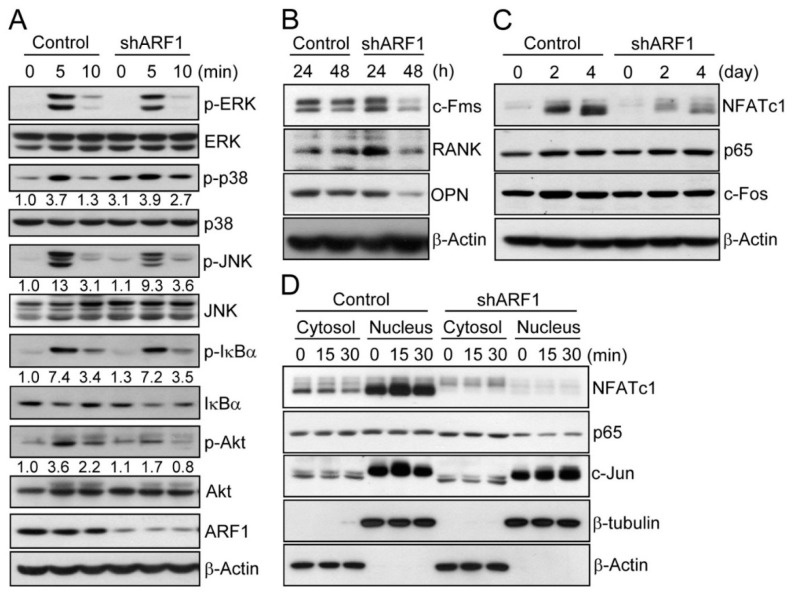
Analysis of osteoclastogenic signals in ARF1-deficient cells. Osteoclast precursors were infected with ARF1 shRNA lentiviral particles or pLKO.1-puro empty control virus particles. (**A**) After cells were treated with RANKL (100 ng/mL) for 0, 5, and 10 min, total or active phosphorylated forms of ERK, p38, JNK, IκBα, and Akt were determined using immunoblot analysis. Fold differences of expression levels were presented; (**B**,**C**) Osteoclast precursors were treated with M-CSF (30 ng/mL) and RANKL (100 ng/mL) for the indicated times and subjected to immunoblot analysis with specific antibodies to c-Fms, RANK, and osteopontin (OPN), NFATc1, p65, and c-Fos; (**D**) Nuclear translocation of NFATc1, p65, and c-Jun was analyzed in the pre-osteoclast stage. After osteoclast precursors were cultured in medium containing M-CSF (30 ng/mL) and RANKL (100 ng/mL) for 2 days, TRAP-positive mononuclear pre-osteoclasts were incubated in the absence of M-CSF and RANKL for 5 h and then treated with RANKL (100 ng/mL) for 0, 15, and 30 min. Cytosolic and nuclear fractions were prepared and subjected to immunoblot analysis. β-Actin and β-tubulin were examined as loading controls. All data are representative of three independent experiments.

To demonstrate that ARF1 is linked to multiple stages of osteoclastogenesis, we analyzed RANKL-induced osteoclastogenic multiple signaling pathways. Upon analyzing transiently activated signal molecules in RANKL-dependent downstream signaling pathways, RANKL treatment to ARF1-deficient cells reduced activation of JNK and Akt as well as prolonging activation of p38 (Figure 4A), but did not affect activation of extracellular signal-regulated kinase (ERK) and IκBα signal molecules. We next measured the expression levels of osteopontin and αvβ3 to evaluate the functional role of ARF1 in osteoclast migration. The levels of endogenous and secreted forms of osteopontin as well as integrin αv (a subunit of αvβ3) were reduced by ARF1 depletion in the early stage of osteoclast differentiation (Figure 4B and Figure S4). The expression levels of M-CSF receptor c-Fms and RANKL receptor RANK responsible for inducing osteoclast proliferation and differentiation, respectively, were also reduced by ARF1 depletion (Figure 4B and Figure S4B). Among osteoclastogenic transcription factors, NFATc1, a master regulator of osteoclast differentiation, was significantly downregulated, whereas p65 (a component of NF-κB) and c-Fos (a component of AP-1) were not altered during differentiation of osteoclasts with ARF1-depletion (Figure 4C and Figure S4B). Further, ARF1-deficient cells showed noticeable reduction of nuclear translocation of NFATc1 (Figure 4D). These results imply that decreased mature osteoclast formation at day 4 post-osteoclastogenesis, as shown in Figure 2B, may be caused by downregulation of NFATc1, which acts as a key player in the late stage of osteoclast formation [10]. Together, our data suggest that ARF1 induces proliferation and migration via M-CSF/c-Fms axis and osteopontin/αvβ3 integrin signaling pathways in the osteoclast precursor stage (Figure S5).

## 3. Discussion

The functional role of the small GTPase ARF1 in osteoclast differentiation remains largely unknown. Our study demonstrates the role of ARF1 in multiple stages of osteoclastogenesis, including proliferation, migration, and fusion. ARF1-knockdown cells showed decreased osteoclast precursor proliferation and migration when compared with control cells. Consistent with our results, depletion of ARF1 has been reported to inhibit proliferation and migration of breast cancer cells [20], indicating that ARF1 is involved in cell proliferation and migration. ARF1 has well documented to play a crucial role in the vesicle-mediated transport, functioning as an important transport regulator [14,21,22]. Cell surface transport of various receptors including angiotensin II type 1 receptor, chemokine receptor 4, α2B-adrenergic receptor, and β2-adrenergic receptor has been shown to be regulated by ARF1. Based on this, we suggest that changes in the level of M-CSF receptor c-Fms and RANKL receptor RANK in ARF1-deficient cells may have resulted from ARF1-mediated alterations of cell surface expression of the receptors. In addition to the role of ARF1 in vesicle-mediated trafficking, ARF1 has known to have functions in the signal transmission from transmembrane proteins [20,23]. ARF1 has been reported to activate the phosphatidylinositol 3-kinase (PI3K) pathway, showing that ARF1 depletion completely abolished the recruitment of the PI3K catalytic subunit to the plasma membrane and inhibited the phosphorylation of Akt [20]. During RANKL-mediated osteoclastogenesis, Akt has been found to induce NFATc1 expression and enhance nuclear localization of NFATc1 through the PI3K/Akt/GSK3β/NFATc1 signaling axis in mouse bone marrow-derived macrophages [24]. In the present study, the reduced activation of Akt in ARF1-deficient cells after RANKL treatment (Figure 4A) may be caused by the failure to recruit the PI3K catalytic subunit to the plasma membrane, and as a result the expression and nuclear localization of NFATc1 was decreased. Dong *et al.* have provided the first evidence indicating that ARF1 plays an important role in the activation of the MAPK signaling pathway [25]. The active form of ARF1 has been shown to be a potent stimulator of ERF1/2 and interaction of ARF1 with α2B-adrenergic receptor via the double Trp motif of the receptor was required for the activation of ERK1/2. ARF6 was reported to activate phospholipase D, ERK1/2, and p38, and interaction of ARF6 with phospholipase D was shown to be essential for those MAPK activation [26,27,28]. Considering the fact that MAPK activation by ARF isoforms such as ARF1 and ARF6 includes interaction of ARF with specific receptors or signaling molecules, the identification of interacting partners of ARF1 should help to explain the mechanism underlying the regulation of MAPKs including p38 and JNK by ARF1 during osteoclastogenesis.

Interestingly, when differentiation of ARF1-depleted osteoclast precursors into multinucleated mature osteoclasts was compared to that of control cells, ARF1 depletion resulted in increased and decreased differentiation of osteoclast precursors into TRAP-positive multinucleated cells on days 3 and 4 after M-CSF and RANKL treatment, respectively. Based on that the number of TRAP(+) MNCs with more than 3 nuclei and a full actin ring at day 3 or day 4 after differentiation of ARF1-depleted cells increased from 47.5 ± 0.70 at day 3 to 652 ± 28.28 at day 4 (Figure S6), the suppressive effect of ARF1 depletion on osteoclast differentiation does not appear to be associated with osteoclast survival. Early maturation during osteoclast formation of ARF1-knockdown cells is thought to be caused by release from cell fusion arrest. This notion is supported by results showing that ARF1-knockdown cells exhibited increased mRNA levels of osteoclast fusion-related genes such as CD44, CD47, E-cadherin, Meltrin-α, and integrin αvβ3 [29,30,31,32,33] and led to higher fusion efficiency of osteoclast precursors than control cells. These results suggest that ARF1 inhibits cell fusion during osteoclast differentiation. Since osteoclast precursor proliferation is stopped after fusion of mononuclear cells [12,13], RANKL-induced ARF1 activity in the early stage of osteoclast differentiation may maintain cell-cell fusion arrest to allow osteoclast precursor proliferation.

Collectively, our data indicate that elevated ARF1 activity in the early stage of osteoclast differentiation resulted in stimulation of cell proliferation and migration as well as suppression of osteoclast fusion, followed by decreased ARF1 activity leading to activation of cell fusion and inhibition of fused cell proliferation and migration. As illustrated in Figure S5, ARF1 promotes osteoclast precursor proliferation via M-CSF/c-Fms signaling pathways, and migration via M-CSF/c-Fms and osteopontin/αvβ3 integrin axis. ARF1 also inhibits cell-cell fusion via suppression of osteoclast fusion-related mediators. In summary, we suggest that ARF1 contributes to the suppression of cell fusion by inhibiting the expression of fusion-related genes, which is required for the cell proliferation and migration in early stage of osteoclast differentiation. In the future, exploration of valuable candidates that can regulate multiple stages of osteoclast differentiation, particularly in cell-cell fusion stages, will provide useful targets to control osteoporotic bone defects due to increased osteoclast differentiation and bone-resorbing function.

## 4. Materials and Methods

### 4.1. Osteoclast Precursor Preparation and Osteoclast Differentiation

BMMs as osteoclast precursors were prepared from the tibia and femur bones of 6-week-old male C57BL/6J mice (Central Lab Animals, Seoul, Korea). Briefly, the bone marrow cavities of long bones were flushed and red blood cells removed using hypotonic buffer containing 0.15 mM NH_4_Cl, 1 mM KCO_3_, and 0.1 mM EDTA, pH 7.4. All animal studies were reviewed and approved by the institutional review board of Yeungnam University Medical Center. Bone marrow cells were incubated in α-MEM containing 10% FBS, antibiotics, and M-CSF (5 ng/mL) for 12 h. Non-adherent monocytes were collected and cultured in α-MEM with M-CSF (30 ng/mL) for 3 days. To induce osteoclast differentiation, osteoclast precursors were treated with M-CSF (30 ng/mL) and RANKL (100 ng/mL) for 4 days with medium exchange every second day. To analyze osteoclast differentiation, the cells were subjected to tartrate-resistant acid phosphatase (TRAP) staining using a leukocyte acid phosphatase staining kit (Sigma-Aldrich, St. Louis, MO, USA). TRAP-positive multinucleated cells (TRAP(+) MNCs) with more than 3 or 10 nuclei were counted using a light microscope.

### 4.2. Quantitative and Semi-Quantitative RT-PCR

Total RNA was prepared from cells with Trizol reagent (Invitrogen, Carlsbad, CA, USA). Total RNA (2 μg) was subjected to reverse transcription into cDNA with oligo dT at 42 °C for 1 h using a M-MLV reverse transcription kit (Invitrogen). For quantitative real-time PCR, we used a SYBR Premix Ex Taq (Takara Bio, Shiga, Japan) on an Applied Biosystems 7500 Sequence Detection System and software (Applied Biosystems, San Francisco, CA, USA). Levels of mRNA expression were analyzed by the comparative Δ threshold cycle method using Gapdh mRNA as the loading control. Semi-quantitative RT-PCR was performed by a Thermo Hybaid PCR Express system (Thermo Hybaid, Ulm, Germany). Primers used are listed in Table S1.

### 4.3. ARF1 Activity Assay

Activation of ARF1 was measured using an Active Arf1 Pull-Down and Detection Kit (Thermo Fisher Scientific, Lafayette, CO, USA) according to the manufacturer’s instructions. Briefly, cells were lysed in ice-cold lysis buffer and spun at 10,000× *g* at 4 °C for 15 min. Glutathione resins and glutathione *S*-transferase (GST)-fusion protein of the GGA3 protein-binding domain were added to each tube, after which the mixture was incubated at 4 °C for 1 h with gentle rocking. After centrifugation, proteins in the resulting pellet were eluted with sodium dodecyl sulfate (SDS) sample buffer. Detection of ARF1-GTP was performed by immunoblot analysis using an anti-ARF1 antibody.

### 4.4. Immunoblot Analysis

For immunoblot assays, osteoclasts were collected, washed with cold PBS, and lysed with lysis buffer consisting of 20 mM Tris-HCl (pH 7.5), 150 mM NaCl, 1% NP-40, 0.5% sodium deoxycholate, 1 mM EDTA, 0.1% SDS, and protease inhibitors (Complete tablets; Roche Molecular Biochemicals, Mannheim, Germany). Cell lysates were then subjected to centrifugation at 10,000× *g* for 10 min at 4 °C. The resulting supernatants were separated on a SDS-polyacrylamide gel electrophoresis (PAGE) and electroblotted onto a nitrocellulose membrane. DC protein assay (Bio-Rad, Hercules, CA, USA) was used to measure protein concentrations. Antibodies against ERK, p-ERK, JNK, p-JNK, p38, p-p38, Akt, p-Akt (Thr308), IκBα, p-IκBα, and RANK were purchased from Cell Signaling Technology. Antibodies against ARF1, c-Fms, NFATc1, p65, c-Fos, osteopontin, c-Jun, β-tubulin, and β-actin were purchased from Santa Cruz Biotechnology. To visualize immune complexes, horseradish peroxidase-conjugated secondary antibodies and ECL reagents (Abfrontier, Seoul, Korea) were used. The level of active (GTP-bound) ARF1 in cells was estimated by an Active ARF1 Pull-Down and Detection Kit (Thermo Fisher Scientific) according to the manufacturer’s instructions.

### 4.5. Knockdown of ARF1 by Short Hairpin RNA (shRNA)

shRNA-mediated knockdown of ARF1 was performed using MISSION Lentiviral Transduction Particles against mouse ARF1 (Sigma, clone ID: TRCN0000100371, TRCN0000100372, TRCN0000100373, and TRCN0000100374) according to manufacturer's instructions. MISSION pLKO.1-puro control transduction particles (Sigma) were used as control virus particles. After BMMs (2 × 10^4^ cells per well in 48-well plates) were infected with shRNA lentiviral particles or pLKO.1-puro empty control particles in the presence of polybrene (8 μg/mL; Sigma) for 12 h, viral particle-containing medium was exchanged for fresh medium and infected cells was selected with puromycin (2 mg/mL) for 2 days. Efficient ARF1 knockdown was validated by RT-PCR and immunoblot analysis. After successful transduction, cells were incubated with M-CSF and/or RANKL for the indicated times.

### 4.6. Proliferation, Migration, and Fusion Assay

Cellular proliferation was analyzed by colorimetric MTT assay. BMMs (2 × 10^4^ cells per well) were plated in 48-well plates and cultured in the presence of 30 ng/mL of M-CSF for the indicated times. After adding MTT solution (Sigma) at a final concentration of 500 μg/mL and 30 min of incubation, DMSO was added. The absorbance was measured spectrophotometrically at 595 nm using a microplate reader (Bio-Rad). Assays for cell migration were performed using Transwell migration chambers (Corning, New York, NY, USA). Osteoclast precursors were starved with M-CSF for 6 h, collected by scrapping, and seeded onto the upper chamber. After adding M-CSF (30 ng/mL) or OPN (100 ng/mL) to the lower chamber, cell migration was examined for 6 h. Non-migrated cells in the upper chamber were removed with a cotton swab. Cells that migrated to the undersides were washed with PBS, fixed with 3.7% formalin, and stained with crystal violet for 1 h. The number of migrated cells was counted under a light microscope. In the fusion assay, when BMMs (2 × 10^5^ cells per well) were seeded in 48-well plates and reached 100% confluence, cells were incubated with M-CSF (30 ng/mL) and RANKL (100 ng/mL) for the indicated times. After fixing with 3.7% formalin, cells were subjected to TRAP staining. The number of TRAP(+) MNCs containing more than 10 nuclei were counted using a light microscope.

### 4.7. Subcellular Fractionation

For nuclear fractionation, cells were lysed in ice-cold lysis buffer consisting of 10 mM Hepes-KOH (pH 7.9), 1.5 mM MgCl_2_, 10 mM KCl, 0.5 mM dithiothreitol, 20 mM sodium pyrophosphate, 10 mM NaF, 1 mM Na_3_VO_4_, and protease inhibitor cocktail. The lysate was centrifuged at 10,000× *g* for 10 min at 4 °C. The supernatant was used as the cytosolic fraction, and the resulting nuclear pellet was suspended in nuclear extraction buffer [20 mM Hepes-KOH (pH 7.9), 25% glycerol, 420 mM NaCl, 1.5 mM MgCl_2_, 0.2 mM EDTA, 0.5 mM dithiothreitol, 20 mM sodium pyrophosphate, 10 mM NaF, 1 mM Na_3_VO_4_, and protease inhibitor cocktail] and centrifuged at 12,000× *g* for 2 min at 4 °C. The resultant supernatant was used as the nuclear fraction.

### 4.8. Statistical Analysis

All quantitative data are presented as the means ± SD from at least three independent experiments. The statistically significant difference between two groups was analyzed using Student's *t*-test. For statistical analysis for multiple comparisons, means between multiple groups were performed using one-way ANOVA analysis with Microsoft 2010 Excel program. A *P* value less than 0.05 was considered statistically significant.

## Supplementary Materials

Supplementary materials can be found at http://www.mdpi.com/1422-0067/16/12/26168/s1.
