# Supplementary material for: ADP-Ribosylation Factor 1 Regulates Proliferation, Migration, and Fusion in Early Stage of Osteoclast Differentiation
Source: Int J Mol Sci. 2015 Dec 9;16(12):29305–14. doi: 10.3390/ijms161226168 (PMC4691111; doi:10.3390/ijms161226168)
Supplement: Supplementary file 1 [file ijms-16-26168-s001.pdf]

Supplementary Materials: ADP-Ribosylation Factor 1 Regulates Proliferation, Migration, and Fusion in Early Stage of Osteoclast Differentiation

Min Jae Kim, Hyunsoo Kim, Seoung Hoon Lee, Dong Ryun Gu, Soo Young Lee, Kyunghee Lee and Daewon Jeong

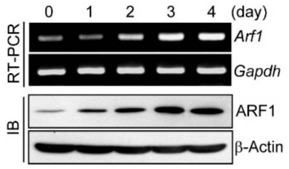

Figure S1. Analysis of ARF1 expression during osteoclast differentiation. The mRNA and protein levels of ARF1 during osteoclast differentiation were determined using RT-PCR and immunoblot analysis (IB). β-actin and *Gapdh* were used as loading controls.

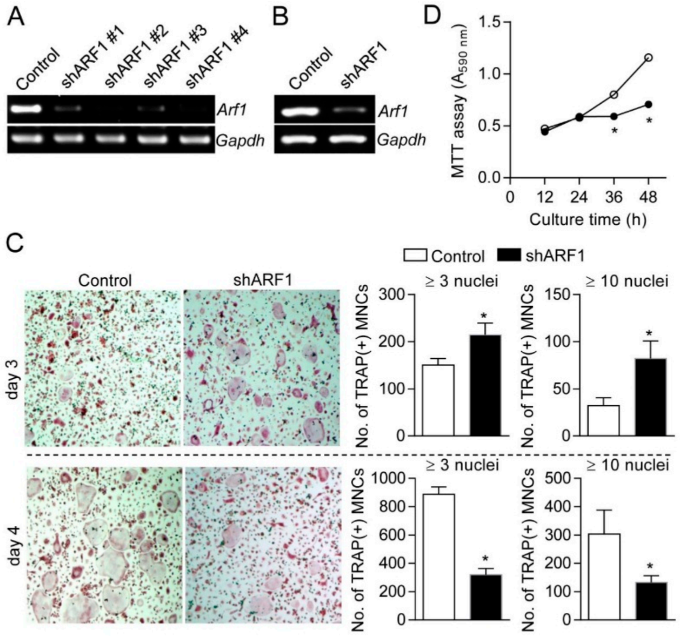

Figure S2. ARF1 knockdown by shRNA in osteoclast precursors and mature osteoclasts. (**A**) Osteoclast precursors were infected with lentiviruses expressing four independent mouse ARF1 shRNAs (#1–#4) or pLKO.1-puro empty control particles as described under "Materials and methods". After 2 days of puromycin selection, the efficacy of shRNA-mediated ARF1 knockdown was determined by RT-PCR analysis. Among them, ARF1 knockdown by shRNA #2 showed the most efficient inhibition of ARF1 expression; (**B**) Osteoclast precursors infected with lentiviruses expressing ARF1 shRNA #2 were selected and further treated with M-CSF (30 ng/mL) and RANKL (100 ng/mL) for 4 days. The mRNA level of ARF1 in mature osteoclasts was determined by RT-PCR analysis. *Gapdh* was used as a loading control; (**C**,**D**) Validation of the effect of ARF1 knockdown on osteoclast precursor differentiation and proliferation using shRNA #4. (**C**) Osteoclast precursors infected with ARF1 shRNA #4 lentiviral particles with 70% confluence (2×10^4 cells per well in 48-well plates) were cultured with M-CSF (30 ng/mL) and RANKL (100 ng/mL) for 3 or 4 days as indicated. TRAP(+) MNCs representing more than 3 or 10 nuclei were counted and photographed using a light microscope. Data are mean ± SD (n = 3) and representative of at least three experiments. * $p < 0.01$; (**D**) Osteoclast precursors infected with ARF1 shRNA #4 lentiviral particles (closed circles) or pLKO.1-puro empty control particles (open circles) were cultured with M-CSF (30 ng/mL) for the indicated times, followed by colorimetric MTT assay. * $p < 0.01$

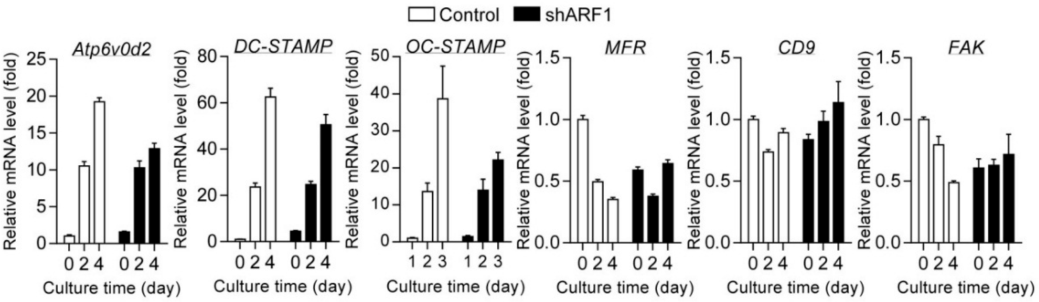

Figure S3. Expression of fusion-related genes in ARF1-depleted osteoclasts. Osteoclast precursors infected with ARF1 shRNA lentiviral particles or pLKO.1-puro empty control particles were cultured with M-CSF (30 ng/mL) and RANKL (100 ng/mL) for the indicated times. Quantitative real-time PCR analysis were used to determine the mRNA expression levels of fusion-related genes such as *Atp6v0d2*, *DC-STAMP*, *OC-STAMP*, *MFR*, *CD9*, and *FAK*. Data are mean ± SD (n = 3).

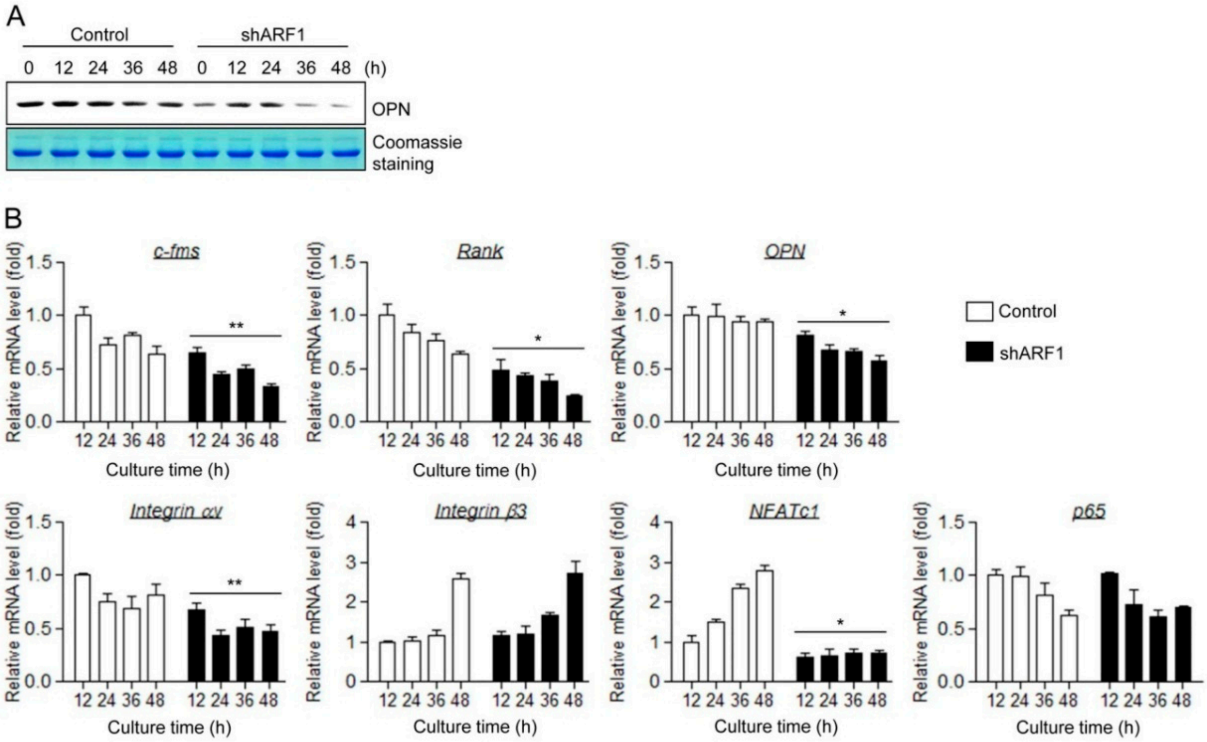

Figure S4. The expression levels of various genes related to osteoclast precursor proliferation and migration in the early stage of osteoclast differentiation. Osteoclast precursors were infected with ARF1 shRNA lentiviral particles or pLKO.1-puro empty control particles. (**A**) OPN level in culture media. Cells were cultured in α-MEM containing 0.5% FBS and treated with M-CSF (30 ng/mL) and RANKL (100 ng/mL) for the indicated times. The conditioned media were collected and the level of secreted OPN was determined using immunoblot analysis. Coomassie blue staining of the gel were presented as a loading control; (**B**) Osteoclast precursors were cultured with M-CSF (30 ng/mL) and RANKL (100 ng/mL) for the indicated times. Quantitative real-time PCR analysis was performed to determine the mRNA expression levels of genes such as *c-fms*, *Rank*, *OPN*, αvβ3, *NFATc1*, and *p65* (a subunit of NF-κB). *Gapdh* mRNA was used as the loading control. The quantitative data between groups were analyzed by one-way ANOVA comparison. * $p < 0.01$, ** $p < 0.05$.

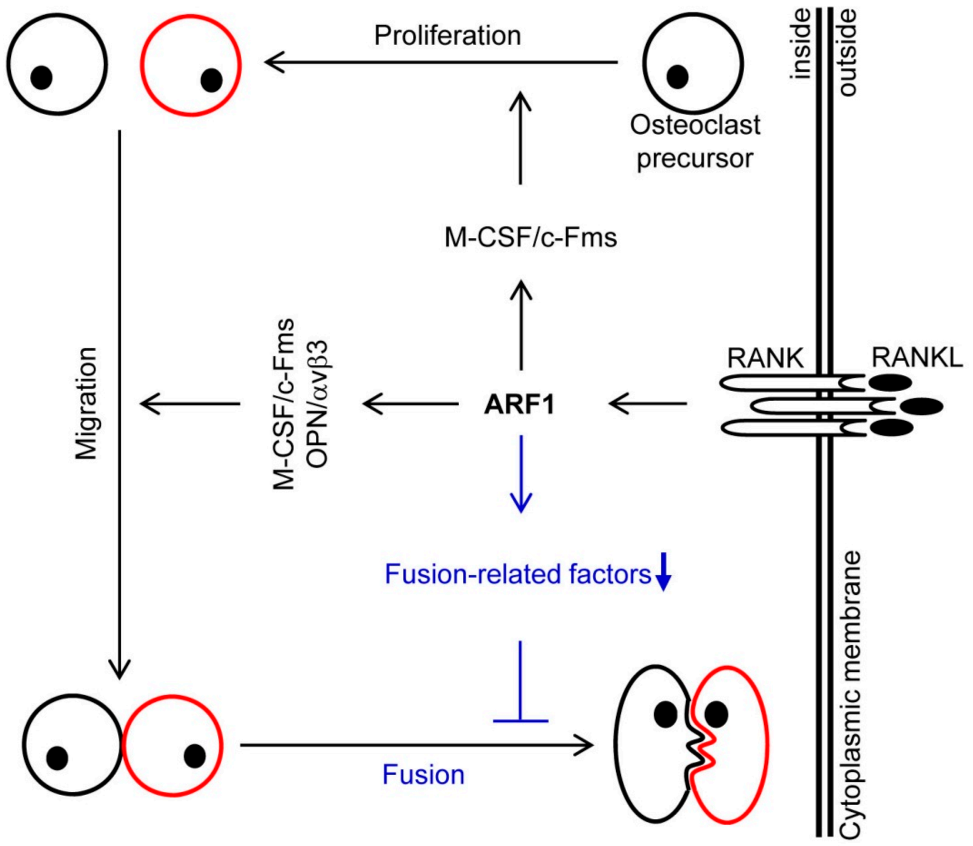

Figure S5. Schematic representation for the functional role of ARF1 in osteoclast differentiation.

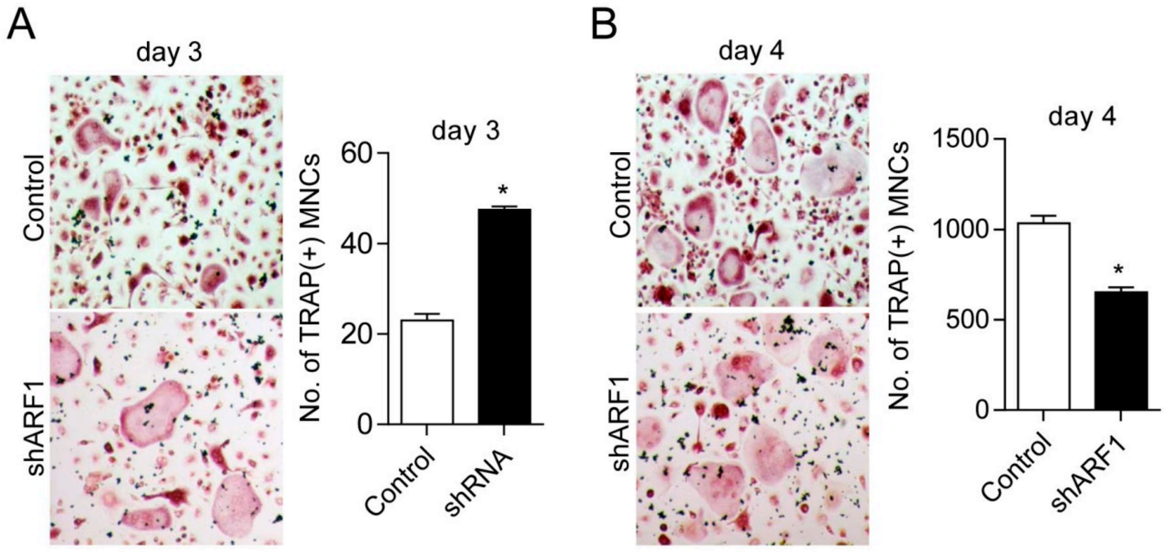

Figure S6. Analysis of TRAP(+) MNCs formation in ARF1-depleted cells during osteoclastogenesis. ARF1-depleted cells were cultured with M-CSF (30 ng/mL) and RANKL (100 ng/mL) for 3 (**A**) or 4 days (**B**) as indicated. TRAP(+) MNCs representing more than 3 nuclei and a full actin ring were counted and photographed using a light microscope. * $p < 0.01$

Table S1. Sequences of PCR primers used in this study.

Gene	Sense (5′→3′)	Antisense (5′→3′)
**Semiquantitative RT-PCR**		
**Gene**	**Sense (5′→3′)**	**Antisense (5′→3′)**
Arf1	TTGGCTGTCTAATCAGCTCC	CCTACACCCAAATCCCTTCT
Gapdh	CAAGGCTGTGGGCAAGGTCA	AGGTGGAAGAGTGGGAGTTGCTG
**Quantitative RT-PCR**		
**Gene**	**Sense (5′→3′)**	**Antisense (5′→3′)**
ATP6v0d2	TCAGATCTCTTCAAGGCTGTGCTG	GTGCCAAATGAGTTCAGAGTGATG
DC-STAMP	TGGAAGTTCACTTGAAACTACGTG	CTCGGTTTCCCGTCAGCCTCTCTC
OC-STAMP	CCTTGGTGCTACAGGCCTAC	CAGAGTCCGAGTTCCCTGTC
E-cadherin	ATGAGCGTGCCCCAGTATCG	TAGCGGCTTCAGAACCACTGC
Meltrin-α,	AAATCCCACGACAATGCTCAGC	CCAGCTCATGTGCCAAGGTCA
CD9	AAGTGCATCAAATACCTGCTCTTC	GCATGCACTGGGACTCTTGTACAG
CD44	GGACTCCAGGGGGAGTTCCCGCAC	CGTCCCATTGCCACCGTTGATCAC
MFR	AAATCAGTGTCTGTTGCTGCTGG	CTGGGGTGACATTACTGATAC
Av	CCTCAGAGAGGGAGATGTTCACAC	AACTGCCAAGATGATCACCCACAC
β3	GATGACATCGAGCAGGTGAAAGAG	CCGGTCATGGATAGTGATGAGTAG
CD47	AGGAGGAGAAAGGAGGTTGC	AACCACGATGACTGTGAGCA
FAK	GGCAGCTGCTTATCTTGACC	TGATGCCCCTGACATCAGTA
c-fms	CAGAGCCCCCACAGATAAAA	GTCCACAGCGTTGAGACTGA
Rank	TTCCCAGTGAAGCAGCAGCCA	GAGATGAACGTGGAGTTACTGTTTC
OPN	CCCATCTCAGAAGCAGAATCT	TTGCTTGGAAGAGTTTCTTGCT
NFATc1	GAGACAGACATCGGGAGGAAGA	GTGGGATGTGAACTCGGAAGA
p65	GCGTACACATTCTGGGGAGT	GTTAATGCTCCTGCGAAAGC
Gapdh	AGGTCGGTGTGAACGGATTTG	TGTAGACCATGTAGTTGAGGT
